# Remimazolam used for sedation during spinal anesthesia puncture provides faster sedation effect and increase recovery rate compared to midazolam: a randomized controlled trial

**DOI:** 10.3389/fphar.2025.1685312

**Published:** 2025-10-20

**Authors:** Peipei Sun, Zhimin Tan, Lianxiong Liang, Jianbo Zhang, Xiaoqiang Li

**Affiliations:** ^1^ Department of Anesthesiology, West China Hospital of Sichuan University, Chengdu, Sichuan, China; ^2^ Department of Anesthesiology, Gansu Provincial Maternal and Child Health Hospital (Gansu Provincial Central Hospital), Chengdu, Gansu, China; ^3^ Department of Anesthesiology, Heze Traditional Chinese Medicine Hospital, Heze, Shandong, China

**Keywords:** remimazolam, midazolam, sedative agents, hemodynamic stability, recovery time

## Abstract

**Background:**

Surgical time-out is essential for patient safety, requiring patients to remain conscious to verify their identity and surgical site. In China’s high-volume operating rooms, this step is crucial to prevent errors. Spinal anesthesia often induces anxiety, making sedation necessary. An ideal sedative must provide effective anxiolysis while allowing rapid recovery. This study compares remimazolam and midazolam to meet these dual demands and assess their cardiorespiratory safety.

**Methods:**

This prospective, randomized controlled trial compared remimazolam and midazolam for sedation in patients undergoing elective ultrasound-guided spinal anesthesia. Patients were assigned to receive either remimazolam (0.1 mg/kg bolus, 0.5–1.0 mg/kg/h infusion) or midazolam (0.07 mg/kg bolus, 0.05–0.1 mg/kg/h infusion). Sedation was titrated to a target Richmond Agitation–Sedation Scale (RASS) score of −4. The primary outcome was the recovery time from RASS –4 to −1. Secondary outcomes included onset time, hemodynamic changes, respiratory parameters, and presences of adverse events.

**Results:**

Of the 103 patients screened, 56 were eligible and randomized into the remimazolam (n = 28) and midazolam (n = 28) groups. The sedation onset time [1.5 (1.0, 2.0) min vs. 3.1 (2.8, 5.0) min, *P* < 0.001] and recovery time [3.9 (3.0, 5.2) min vs. 6.1 (3.1,10.0) min, *P* = 0.0016] was significantly shorter in the remimazolam group than midazolam group. Remimazolam had no significant impact on procedural time of ultrasound-guided spinal anesthesia. Both groups showed significant within-group decreases in mean arterial pressure during sedation (remimazolam: from 81.5 to 75 mmHg; midazolam: from 81.7 to 72.4 mmHg; *P* < 0.05), with no significant inter-group difference. At end of the procedure, remimazolam group had a higher respiratory rate than midazolam group (18.6 ± 3.3 vs. 16.8 ± 2.8) (*P* < 0.05). No significant differences were observed in heart rate, end-tidal carbon dioxide partial pressure, and oxygen saturation between groups. Remimazolam was not associated with an increased risk of adverse events.

**Conclusion:**

Remimazolam demonstrated superior recovery in sedation depth, with a significantly shorter time to regain consciousness (RASS –4 to −1), compared with midazolam in patients undergoing ultrasound-guided spinal anesthesia. No significant differences in circulatory or respiratory parameters were observed between groups, though the study may lack power to detect minor safety effects.

## 1 Introduction

Subarachnoid block (spinal anesthesia), a form of neuraxial anesthesia, which involves injection of local anesthetics into the cerebrospinal fluid via the lumbar spine to anesthetize spinal nerves. It is widely used for anesthesia in a variety of lower limbs, lower abdomen, pelvic and perineal operations. Ultrasound-guided spinal anesthesia allows real-time visualization of ultrasound images to adjust needle trajectory, which improves the accuracy of intervertebral space positioning, increases the success rate of spinal puncture, reduces procedure-related complications, and minimizes patient discomfort (Grau et al., 2004).

However, there are still some challenges in the clinical application of spinal anesthesia. Skilled ultrasound guidance reduces the risk of repeated punctures compared to traditional blind techniques, yet patients may still experience anxiety, fear, and discomfort. More importantly, the high precision required may prolong the procedure time, further exacerbating patient discomfort. Overly anxious or restless patients may struggle to tolerate the procedure, potentially compromising the stability of the anesthetic effect and the smooth progression of surgery. Effective sedation is essential to alleviate patient anxiety, it reduces procedural risks, enhances patient comfort tolerance and overall surgery satisfaction. Once benzodiazepines are administered for preoperative sedation, patients may not regain full alertness within a short period of time. If a preoperative time-out is required during this window, they may be unable to verify their identity or the surgical site. This delay in recovery could compromise the safety and quality of the surgery.

Current evidence shows that midazolam, a conventional benzodiazepine, effectively relieves preoperative anxiety but is metabolized by hepatic CYP3A enzymes, with an elimination half-life of 1.5–4.3 h (Prommer, 2020; Zaporowska-Stachowiak et al., 2019). Clearance may be prolonged in hepatic or renal impairment. Its use is associated with respiratory and cardiovascular depression, residual sedation, and delayed neurocognitive recovery—factors that can impair responsiveness during the preoperative time-out, particularly with spinal anesthesia, thereby hindering anesthetic assessment and surgical workflow (Reves et al., 1985; Forster et al., 1980). In contrast, remimazolam is a novel ultra–short-acting benzodiazepine with rapid onset, controllable duration, and minimal hemodynamic effects. It has a distribution half-life of 0.5–2 min and a terminal elimination half-life of 7–11 min (Petersen et al., 2024; Zhou et al., 2017), undergoing rapid hydrolysis by nonspecific tissue esterases to an inactive metabolite. Metabolism is independent of hepatic and renal function, with negligible risk of accumulation, enabling effective sedation while permitting dynamic intraoperative assessment of consciousness (Lee and Shirley, 2021; Kim and Fechner, 2022).

Notably, no prior studies have investigated its application for sedation during ultrasound-guided spinal anesthesia puncture. This prospective randomized controlled trial aims to compare the sedative efficacy and safety of remimazolam *versus* midazolam during the ultrasound-guided spinal puncture, so as to provide reference for clinical practice.

## 2 Materials and methods

### 2.1 Study design and participants

This prospective, randomized study was conducted at West China Hospital, Sichuan University from November 2024 to March 2025. The inclusion criteria were patients aged ≥18 years, scheduled to undergo ultrasound-guided spinal anesthesia for elective surgery, who were capable of understanding the study protocol and had the ability to participate in the study, and who agreed to sign the informed consent form. All participants underwent minor, low-risk procedures, including mixed hemorrhoids, perianal abscesses, anal fissures, and sacrococcygeal or epidermal cysts. These surgeries are brief, minimally invasive, and involve little intraoperative bleeding, making them suitable for evaluating the effects of sedatives on intraoperative conditions and recovery. Most patients were young or middle-aged adults with good baseline health and few chronic comorbidities, minimizing confounding from organ dysfunction, altered drug metabolism, or procedural complexity.

Exclusion criteria were based on the American Society of Regional Anesthesia (ASRA) and Pain Medicine Evidence-Based Guidelines (fifth Edition), ASRA Infection Control Consensus, and the 2023 guidelines from the American Society of Anesthesiologists (ASA), aiming to exclude patients whose conditions may compromise anesthesia safety or efficacy (Kopp et al., 2025; Provenzano et al., 2025; Vallejo et al., 2024). The study ultimately excluded patients with severe hepatic or renal dysfunction, severe respiratory or circulatory diseases, coagulation disorders, cachexia, myasthenia gravis, schizophrenia, severe depression, sleep apnea syndrome, recent anticoagulants usage, allergy to local anesthetics or study drugs, and those who had an infection at the puncture site.

Ethical approval for this study was provided by the Institutional Review Board on 8 October 2024. The trial was registered prior to patient enrolment at the Clinical Research Information Service (https://www.medicalresearch.org.cn). The protocol of this study was based on the ethical standards of the Declaration of Helsinki. The trial registration number is ChiCTR2400092466. We obtained written informed consent from each patient before their participation.

### 2.2 Randomization and blinding method

This study was a double-blinded, randomized, controlled trial, with patients randomly assigned in a 1:1 ratio to either the midazolam or remimazolam group using a computer-generated random number table. Throughout the study, both patients and recorders (including data analysts) were blinded to the group allocation. An individual blinded investigator was responsible for each preoperative screening, obtained informed consent, and recorded data. Another unblinded research assistant, who did not participate in subsequent observation or data collection, prepared the drugs according to the randomization and assisted the anesthesiologist in initiating the infusion. The bolus dose was determined based on the package insert, relevant literature, and pilot testing to achieve a target RASS score of −4. The maintenance dose was administered within the recommended range of the drug label and titrated according to the patient’s RASS score. Anesthesiologist involved in the study had over 5 years of clinical experience and had undergone standardized training in the use of study medications. To ensure consistency and blinding, the drug packaging was sealed, identical infusion pumps were used, and both the total volume and infusion rate were adjusted according to clinical requirements (surgical type, anesthetic duration, incision type, and patient condition). Infusion volumes were automatically recorded by the pumps and analyzed by blinded investigators. To ensure patient safety, the anesthesiologist was not blinded, and all procedures were performed by the same anesthesiologist skilled in ultrasound-guided spinal anesthesia. The anesthesiologist needed to know the drug allocation to adjust anesthesia based on the patients’ response but had no access to any other group-related data, thus preserving the integrity of the blinding.

### 2.3 Outcome measures

Richmond agitation-sedation scale (RASS) was used to assess the depth of sedation in this study. The RASS consists of 10 levels of sedation/agitation: five levels of sedation, one of calm alertness and four levels of agitation (Sessler et al., 2002). Sedation and calm alertness scores are shown in Table 1.

**TABLE 1:** Richmond agitation-sedation scale and corresponding descriptions.

Score	Term	Description
0	Alert and calm	-
−1	Drowsy	Not fully alert, sustained (>10 s) awareness with eye contact to voice
−2	Light sedation	Awakens briefly (<10 s) with eye contact to voice
−3	Moderate sedation	Movement (but no eye contact) to voice
−4	Deep sedation	No response to voice, but eye opens or movement to physical stimulation
−5	Unarousable	No response to voice or physical stimulation

**FIGURE 1:**
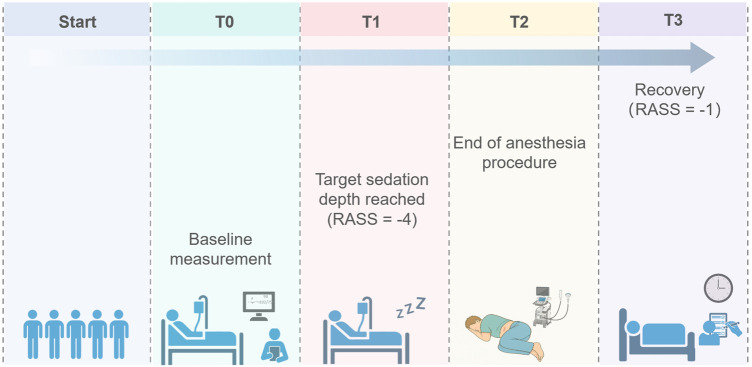
Schematic diagram of data collection points. T0, the time point for baseline measurement; T1, the time to reach the target RASS score of −4; T2, the time marking the end of the anesthesia procedure; and T3, the time when the RASS score recovered to −1.

Sedation level was evaluated at 1 min intervals, with continuous stopwatch timed to obtain second-level accuracy. In particular, we focused on the following key time points: T0, the time point for baseline measurement; T1, the time to reach the target RASS score of −4; T2, the time marking the end of the anesthesia procedure; and T3, the time when the RASS score recovered to −1, at which both consciousness status and vital signs were assessed (Figure 1). The recovery time was used as the primary outcome in this study, defined as the time from RASS-4 to RASS-1 of the patient following the drug withdrawal. Secondary outcomes included the sedation onset time, defined as the time from awake to RASS-4 after drug infusion; hemodynamic and respiratory parameters, including mean arterial pressure (MAP), heart rate (HR), respiratory rate (RR), peripheral capillary oxygen saturation (SpO_2_), and end-tidal carbon dioxide (EtCO_2_) changes. Additionally, we monitored and recorded several adverse events that could occur during sedation and recovery, including allergic reactions, hypoxemia, decrease in MAP, use of vasopressors, nausea, vomiting, hiccups, and nerve-related complications.

### 2.4 Anesthesia and sedation protocol

After the patient was verified, the peripheral venous way of the upper limbs was set, ETCO_2_ monitoring by nasal tube and a 2 L/min oxygen inhalation was induced in the same way. The five-lead electrocardiography, non-invasive blood pressure, oxygen saturation were routinely monitored. The patient was placed in the left lateral decubitus position with flexed knees and neck. After sterile skin preparation and draping, ultrasound-guided spinal anesthesia was performed. The anesthesiologist utilized the Mindray M9 ultrasound system equipped with a C5-1S probe and performed the procedure under sterile conditions. Scanning was initiated from the sacrum in a paramedian sagittal oblique plane, sliding the probe cephalad to identify the L3-4 interlaminar space. Structures such as the ligamentum flavum and anterior and posterior complexes became visible on the ultrasonic images. Using an in-plane needle insertion technique, the anesthesiologist dynamically monitored the needle’s trajectory and depth in real-time. Upon reaching the subarachnoid space, the stylet was withdrawn, confirming free flow of cerebrospinal fluid (CSF). The needle bevel was oriented caudally, and 2.5 mL of ropivacaine hydrochloride injection was administered slowly. Following medication delivery, the needle was withdrawn, and the patient was assisted into a supine position.

After the patient was positioned, remimazolam (Remimazolam Besylate for Injection; Yichang Humanwell Pharmaceutical) was administered as a single dose of 0.1 mg/kg followed by a continuous infusion at a rate of 0.5–1.0 mg/kg/h via an infusion pump. The infusion rate was adjusted to achieve the RASS score of −4 where the procedure was started and stopped once it completed. In the midazolam (Jiangsu Nhwa Pharmaceutical) group, bolus dose of 0.07 mg/kg was intravenously injected and then 0.05–0.1 mg/kg/h was continuously infused to achieve the same target sedation level until the spina anesthesia was finished.

During the entire implementation of the protocol, face masks and a Jackson-Rees modified breathing circuit were readily available. If SpO_2_ decreased to 90% or less, the anesthesiologist would immediately perform jaw thrust, increase oxygen flow rate, or give face mask ventilation as needed.

During the performance of subarachnoid block, if the patient had body movement, the anesthesia assistant must help maintain proper positioning. Management varied depending on the situation: 1) If movement occurred due to pain during subcutaneous local anesthetic infiltration, adequate local anesthesia would typically resolve the movement in most patients. 2) If movement occurred due to the spinal needle having contacted the nerve root, the needle should be withdrawn immediately, and the trajectory would be adjusted. Intraoperative MAP sustained below 60 mmHg for ≥5 min or an acute drop to <50 mmHg triggered immediate pharmacologic intervention. Given that vasopressors intrinsically modulate HR, we coupled the choice of agent to concurrent HR: ephedrine 5 mg intravenously for HR lower than 60 bpm, phenylephrine 30–50 μg intravenously for HR ≥ 60 bpm. Because the primary objective was to evaluate the global hemodynamic stability conferred by the sedation regimen, HR changes attributable to vasopressor therapy were excluded from subsequent statistical analyses.

### 2.5 Sample size calculation

The primary endpoint was the time required for patients to recover from RASS-4 to RASS-1 after stopping infusion of two groups. According to previous studies, the recovery time from sedation in the remimazolam group ranged from 6 to 15.1 min (Pastis et al., 2019; Guo et al., 2022; Lu et al., 2022), while in the midazolam group it ranged from 11.5 to 15.2 min (Pambianco et al., 2016; Rex et al., 2018; Borkett et al., 2015). A superiority margin of Δ = 2 min, a combined standard deviation of σ = 1.8 min, a two-sided significance level of α = 0.05, and a statistical power of 95% were set. A 10% dropout rate was assumed to account for potential losses to follow-up.

Based on the sample size calculation formula, 24 participants per group were required, for a total sample size of 48 participants. Ultimately, 28 participants were enrolled in each group, with a total of 56 participants.

$$n=\frac{2\sigma^{2}{\left(Z_{1-\alpha /2}+Z_{1-\beta }\right)}^{2}}{\Delta^{2}}$$

### 2.6 Statistical analysis

Continuous variables are expressed as mean ± SD for normally distributed data or as median (Q1, Q3) for non-normally distributed data. Normality of the data was assessed using the Kolmogorov-Smirnov test. For data that follow a normal distribution, the independent t-test was used to compare means between groups. For non-normally distributed data, the Mann-Whitney U test was applied to compare medians between groups. All tests were two-sided, and *P*-values <0.05 were considered statistically significant. All analyses were performed using SPSS^®^ 27.0 (IBM^®^ Corp.) and GraphPad Prism 10.3.1 software.

## 3 Results

### 3.1 Baseline characteristics

**FIGURE 2:**
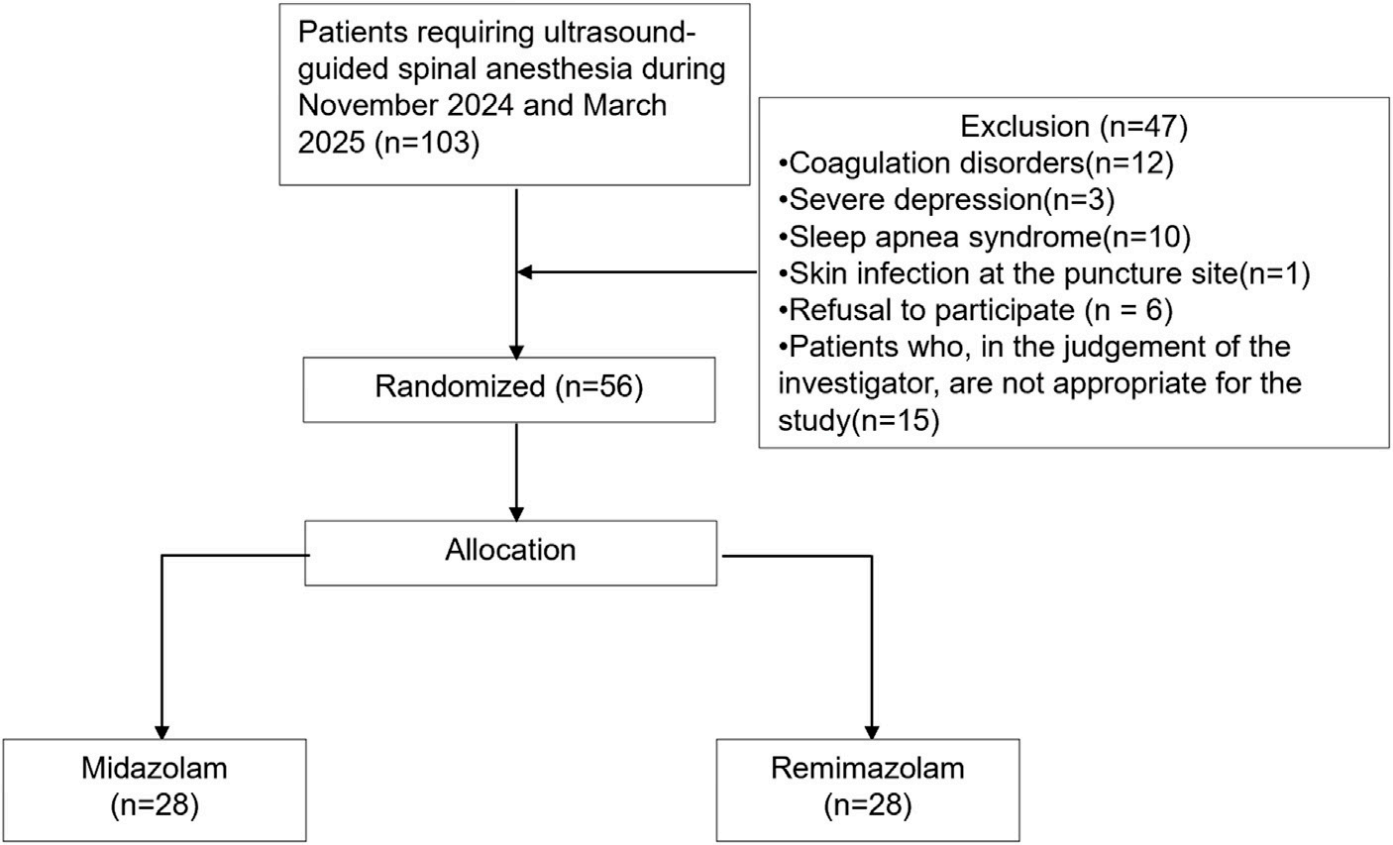
Consolidated standards of trials.

A total of 103 patients were screened for eligibility. Of these, 47 were excluded: 26 (55.3%) due to contraindications (e.g., coagulopathy, severe depression, obstructive sleep apnea, or infection at the puncture site), 6 declined to participate, and 15 were excluded at the discretion of the investigators (Supplementary Table 1). 56 were eligible and randomly assigned to either the midazolam (n = 28) or remimazolam (n = 28) group (Figure 2). All patients received their allocated intervention. The enrolled population was predominantly composed of young and middle-aged adults, with only two patients over 65 years of age. The mean age was 39.5 years, and 34% of participants were male. There were no significant differences between groups in body mass index (BMI), baseline blood pressure, heart rate, oxygen saturation, or end-tidal carbon dioxide levels. The procedure time of spinal anesthesia was comparable between the groups (P = 0.21). The mean surgical duration was 94.4 min, and the average intraoperative fluid volume was 2.1 mL/kg/h, with no significant differences observed between groups (Table 2).

**TABLE 2:** Comparison of patient characteristics.

Variables	Remimazolam group (n = 28)	Midazolam group (n = 28)	*P*-value
Sex (M/F)	10/18	9/19	0.78
Age (yr)	41.0 ± 11.9	38.0 ± 10.8	0.33
Height (cm)	161.0 (157.0, 170.0)	164.0 (160.0, 168.0)	0.70
Weight (kg)	56.0 (50.0, 69.0)	60.8 (57.5, 67.5)	0.23
Body mass index (kg/m^2^)	21.5 (19.7, 23.1)	22.5 (21.2, 24.8)	0.12
Baseline SAP (mmHg)	116.6 ± 14.9	115.6 ± 14.4	0.80
Baseline DAP (mmHg)	67.8 ± 8.2	69.9 ± 10.6	0.40
Baseline MAP (mmHg)	81.5 ± 11.4	81.7 ± 10.4	0.95
Baseline HR (bpm)	76.1 ± 10.8	81.9 ± 15.6	0.11
Baseline SpO_2_ (%)	98.0 ± 1.5	97.8 ± 1.9	0.64
Baseline, ETCO_2_ (mmHg)	36.6 ± 4.2	36.0 ± 5.8	0.62
Baseline RR (br/min)	17.2 ± 4.2	17 ± 4.5	0.88
Duration of procedure time (min)	2.9 (1.9, 4.3)	2.1 (2.0, 3.3)	0.21
Surgical Duration (min)	92.5 (82.8, 104.3)	96.0 (89.8, 101.0)	0.88
Intraoperative Fluid Volume (mL/kg/h)	2.1 (2.0, 2.1)	2.1 (2.0, 2.2)	0.82

Data are presented as median (Q1, Q3) for non-normally distributed variables, or mean ± standard deviation for normally distributed variables. *P*-values were derived from the independent t-test for normally distributed data or referenced the Mann-Whitney U test for non-normally distributed data. SAP, systolic arterial pressure; DAP, diastolic arterial pressure; MAP, mean arterial pressure; HR, heart rate; SpO2, oxygen saturation; ETCO_2_, the end-tidal carbon dioxide partial pressure; RR, respiratory rate.

### 3.2 Recovery and onset times

**FIGURE 3:**
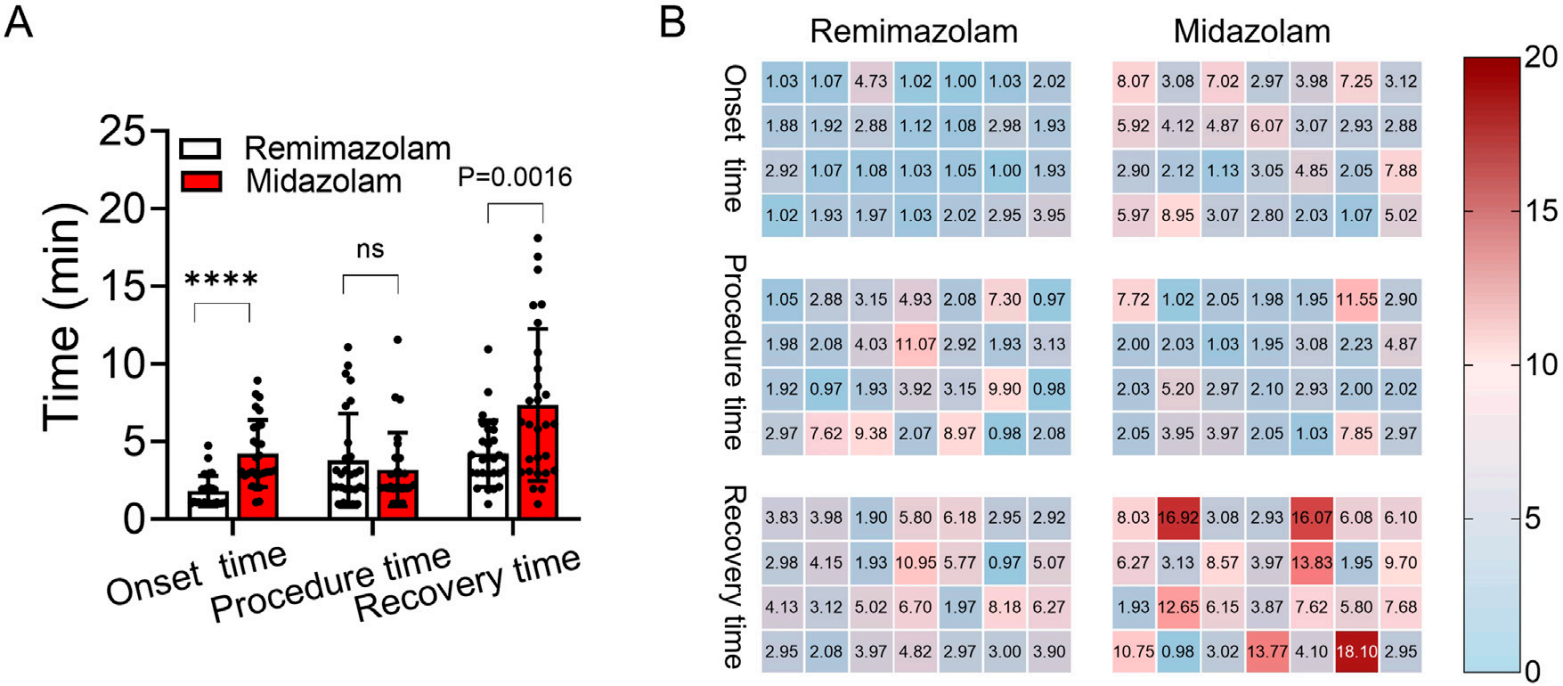
Comparison of onset, procedure, and recovery times between remimazolam and midazolam groups. **(A)** Chart comparison of the onset, procedure, and recovery time between the remimazolam group (white) and the midazolam group (red). **(B)** Heatmap of onset, procedure, and recovery time (min) differences between remimazolam and midazolam groups. ****: *P* < 0.01; ns: no significant difference.

The recovery time was significantly shorter in the remimazolam group than which in the midazolam group [3.9 (3.0, 5.2) min vs. 6.1 (3.1, 10.0) min, *P* = 0.0016] (Figure 3A). Additionally, the time to achieve RASS-4 was significantly shorter in the remimazolam group than in the midazolam group [1.5 (1.0, 2.0) min vs. 3.1 (2.8, 5.0) min, *P* < 0.01]. The heatmap clearly visualizes that midazolam prolongs onset and recovery time compared to remimazolam, with no significant difference in procedure time (Figure 3B).

### 3.3 Hemodynamic, respiratory and basic vital signs during sedation

**FIGURE 4:**
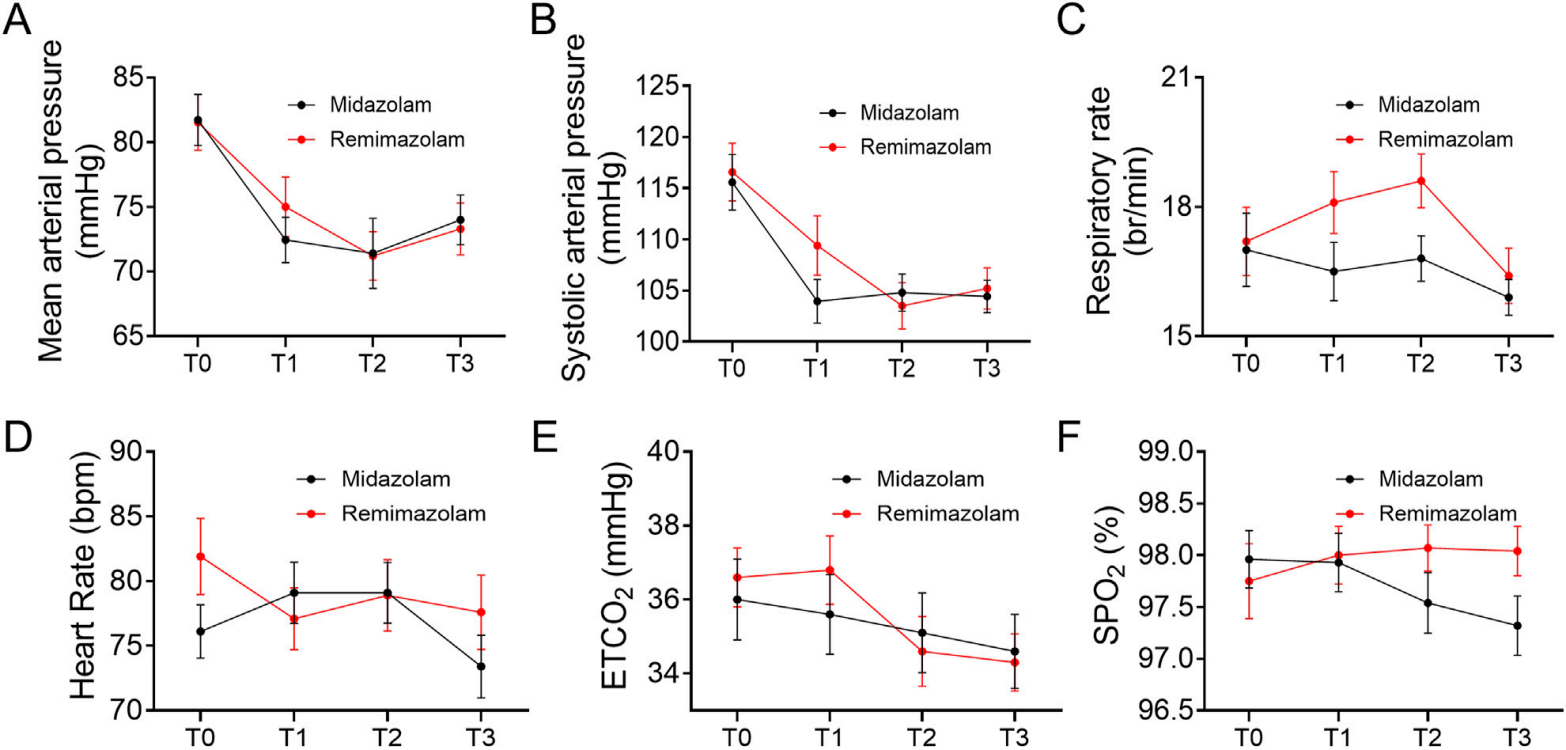
Trend of vital parameters at different time points (T0, T1, T2, T3). **(A)** Trend of mean arterial pressure at different time points. **(B)** Trend of systolic blood pressure at different time points. **(C)** Trend of respiratory rate at different time points. **(D)** Trend of heart rate at different time points. **(E)** Trend of end-tidal carbon dioxide at different time points. **(F)** Trend of oxygen saturation at different time points. Each subfigure illustrates the changes in the respective parameters at baseline (T0), when the RASS target score reached −4 (T1), at the end of the anesthesia procedure (T2), and when the RASS score recovered to −1 (T3). SpO2: oxygen saturation, ETCO_2_: the end-tidal carbon dioxide partial pressure.

During the sedation period, mean arterial pressure, blood pressure, heart rate, respiratory rate, oxygen saturation, ETCO_2_ values and sedation depth were monitored per minute. A decrease was observed in mean arterial pressure and systolic arterial pressure as shown in Figures 4A,B. In intra-group evaluations, it was observed that mean arterial pressure values in the two groups were significantly lower than the baseline (*P*  < 0 0.05). However, the mean arterial pressure values during the study period did not differ between the groups (Table 3). The heart rate values between groups did not differ (Figure 4D). At the time of RASS score recovery to −1 (T3), ETCO_2_ values in the remimazolam group was significantly lower than the baseline (*P*  < 0 0.05). The oxygen saturation, ETCO_2_ values did not differ between the groups during the study period (Figures 4E,F). The nasal CO_2_ monitoring catheter used in this study has an oxygen delivery function, which may have resulted in CO_2_ dilution and thus created a discrepancy between the measured and actual values. However, respiratory rate monitoring remained accurate. At the time to reach RASS target score −4 (T1) and the end of anesthesia procedure (T2), the respiratory rate of remimazolam group increased, while in the midazolam group, it decreased, though no significant difference from baseline was observed (Figure 4C). At T2, the respiratory rate in the midazolam group was significantly lower than in the remimazolam group (*P* < 0.05).

**TABLE 3:** Comparison of hemodynamic and respiratory parameters within and between groups during the study period.

Parameters	MAP (mmHg)			HR (bpm)			RR (br/min)			ETCO_2_ (mmHg)			SpO_2_ (%)		
Groups	R	M	*P*	R	M	*P*	R	M	*P*	R	M	*P*	R	M	*P*
T0	81.5 ± 11.4	81.7 ± 10.4	0.95	76.1 ± 10.8	81.9 ± 15.6	0.11	17.2 ± 4.2	17 ± 4.5	0.95	36.6 ± 4.2	36.0 ± 5.8	0.62	98.0 ± 1.5	97.8 ± 1.9	0.64
T1	75 ± 12.2*	72.4 ± 9.3*	0.39	79.1 ± 12.5	77.1 ± 12.7	0.54	18.1 ± 3.8	16.5 ± 3.6	0.12	36.8 ± 4.9	35.6 ± 5.7	0.42	97.9 ± 1.5	98.0 ± 1.5	0.86
T2	71.2 ± 9.9*	71.4 ± 14.4*	0.94	79.1 ± 12.4	78.9 ± 14.6	0.95	18.6 ± 3.3	16.8 ± 2.8	0.03	34.6 ± 5.0	35.1 ± 5.7	0.69	97.5 ± 1.6	98.1 ± 1.2	0.15
T3	73.3 ± 10.6*	74.0 ± 10.2*	0.79	73.4 ± 2.8	77.6 ± 15.2	0.28	16.4 ± 3.4	15.9 ± 2.2	0.49	34.3 ± 4.1*	34.6 ± 5.3	0.80	97.3 ± 1.5	98.0 ± 1.3	0.06

Data are presented as median (Q1, Q3) for non-normally distributed variables, or mean ± standard deviation for normally distributed variables. Values are presented as mean ± SD. R, remimazolam; M, midazolam; MAP, mean arterial pressure; HR, heart rate; RR, respiratory rate; ETCO2, the end-tidal carbon dioxide partial pressure; SpO2, oxygen saturation. T0: the time point for baseline measurement, T1: the time to reach RASS, target score −4, T2: the time of the end of anesthesia procedure, T3: the time of RASS, score recovery to −1. *P value was statistically significant.

### 3.4 Adverse events and safety profile

No significant difference was observed in general adverse events between the two groups (Table 4). Nausea, vomiting, allergic reaction and hypoxemia rates were similar for both groups (*P* > 0.05). The number of patients who required vasoactive drugs and the percent MAP decrement was not different between the two groups. Hiccup occurred in 1/28 (3.6%) patient in both groups, with no difference of the incidence. In addition, none of the patients with ultrasound-guided spinal anesthesia under sedation experienced nerve-related complications.

**TABLE 4:** Incidence of complications in the remimazolam and midazolam groups.

Complications	Remimazolam group (n = 28)	Midazolam group (n = 28)	*P*-value
Allergy	0 (0.0%)	0 (0.0%)	1.00
Hypoxemia	0 (0.0%)	0 (0.0%)	1.00
MAP decrement	17.6 ± 9.7	19.8 ± 9.0	0.55
Use of vasopressor	0 (0.0%)	0 (0.0%)	1.00
Nausea	0 (0.0%)	0 (0.0%)	1.00
Vomiting	0 (0.0%)	0 (0.0%)	1.00
Hiccup	1 (3.6%)	1 (3.6%)	1.00
Nerve-related complications	0 (0.0%)	0 (0.0%)	1.00

Values are presented as number (%) or mean ± SD., The MAP decrement was calculated as the percentage difference between the baseline MAP, measured in the anesthesia preparation room and the lowest MAP, during the study period.

## 4 Discussions

### 4.1 Main finding

During invasive procedures like nerve block and spinal puncture, patients often experience significant anxiety. Preoperative administration of benzodiazepines could improve the perioperative experience. However, prior to surgery, patient identification and surgical site verification are required, and the patient’s RASS score must be at least −1. For short procedures, it is essential that the patient regains the ability to respond before surgery begins. Therefore, an ideal sedative should act rapidly and allow for quick recovery upon discontinuation. In this study, we compared the efficacy and safety of remimazolam and midazolam as sedatives for ultrasound-guided spinal anesthesia. Remimazolam showed shorter onset time and recovery time after the end of the procedure than midazolam. Its hemodynamic effects were comparable to those of midazolam. No respiratory events requiring intervention were observed during sedation. Side effects associated with the drug were acceptable and easy to manage. However, given the relatively small sample size and the fact that safety was not the primary endpoint, these findings should be interpreted with caution.

### 4.2 Current evidence

The major findings of our study were the rapid sedation and wakefulness induced by remimazolam. These results are consistent with a previous published clinical trial by Kim SH et al., in which compared with midazolam, remimazolam has a shorter onset time [2 (1, 4) min vs. 3 (2, 5) min, *P* = 0.006] and recovery time [2 (1, 5) min vs. 5 (1, 12) min, *P* = 0.035] during bronchoscopy (Morimoto, 2022). These results correlate with the pharmacokinetic advantages of remimazolam. As it is rapidly converted into an inactive metabolite, it is characterized by rapid onset and swift recovery from sedation (Sneyd, 2023). Because of the rapid recovery of consciousness during anesthesia preparation room, transfer to the operating room was swift.

### 4.3 Clinical significance

The MAP decrease during the study period does not differ significantly between the two groups and vasoactive drugs were not required. These findings suggest that post-sedation hypotension in the remimazolam group were mild and effects resolved without having to use vasoactive drugs. However, the sample size was limited, and the study was not powered to detect severe or rare adverse events. Notably, multiple previous studies have consistently shown that remimazolam preserves hemodynamic parameters even at higher doses, indicating a broad therapeutic index (10–12).

Benzodiazepines, in general, exhibit a more favorable respiratory profile compared to propofol. Among benzodiazepines, remimazolam has shown minimal respiratory compromise in adults (Barbosa et al., 2024; Ahmer et al., 2024; Hansen and Engelhardt, 2025; Pereira et al., 2024). In our study, the respiratory rate of midazolam group was lower than the remimazolam group at the time of T2, which is consistent with the aforementioned conclusions, indicated that remimazolam had less inhibitory effect on respiration. Hypopnea and apnea were both absent the whole time for the two groups.

Our study targeted a sedation depth of RASS -4 rather than −5 to preserve the patient’s ability to react to pain or nerve root stimulation, thereby reducing the risk of nerve injury. Additionally, during spinal anesthesia, patients typically needed to cooperate with the surgeon to ensure accurate operation, making sedation a critical tool to ensure both patient comfort and the smooth progression of the procedure. The duration of recovery directly impacts the patient’s postoperative recovery and the overall efficiency of the surgery. Prolonged recovery time increases the likelihood of risk exposure, while a shorter recovery time not only reduces post-anesthesia discomfort but also improves postoperative patient satisfaction, lowers healthcare costs, and enhances hospital resource utilization.

To improve the reliability and safety of the procedure, real-time ultrasound-guided spinal puncture technology was used to accurately visualize the relative position of the puncture path and surrounding anatomical structures (Chong et al., 2017; Elsharkawy et al., 2017). A narrative review by Pierson D.et al. also mentioned that real-time ultrasound guided spinal anesthesia could be the future of safer and more effective spinal anesthesia. In the preliminary phase of this study, we performed ultrasound-guided spinal anesthesia on fully awake patients and most of the patients complained of significant discomfort when subcutaneous local infiltration occurred with no notable adverse effects. Importantly, no nerve-related complications occurred.

This study has several limitations. First, it was a single-center trial with a relatively small sample size, which may limit the generalizability of the findings. The limited sample size may also be insufficient to detect infrequent or severe adverse events. For example, although no cases of hypopnea or apnea were observed in either group, the relatively small cohort and short observation window does not rule out the possibility of rare respiratory or other complications. Secondly, this study did not employ standardized tools to assess anxiety before or after spinal anesthesia. Although patients with severe depression were excluded at enrollment, the lack of quantitative anxiety data limits interpretation of how psychological state may affect sedative response. Future studies should incorporate validated anxiety and depression scales to better evaluate differential drug effects across psychological profiles. Finally, given that the surgical procedures involved in this study were short in duration and minimally invasive, the enrolled population mainly consisted of young to middle-aged adults, despite our initial intention to include all patients aged ≥18 years. Only two participants were older than 65 years (aged 67 and 68). Therefore, the generalizability of our findings to elderly, pediatric, or high-risk populations remains limited. Further post-marketing surveillance and real-world studies are warranted to validate the safety and efficacy of remimazolam in broader clinical settings.

## 5 Conclusion

Remimazolam, as a sedative for ultrasound-guided spinal anesthesia, demonstrated a shorter onset and recovery time, offering higher operational efficiency and patient comfort compared to midazolam. Its safety profile was favorable, with no significant hemodynamic or respiratory adverse reactions, further confirming its suitability for this application. Given remimazolam’s pharmacokinetic advantages, rapid recovery characteristics, and minimal side effects, it is expected to become an effective agent for short-term procedural sedation, enhancing both clinical practice and patient satisfaction in spinal anesthesia.

## Data Availability

The original contributions presented in the study are included in the article/Supplementary Material, further inquiries can be directed to the corresponding author.

## Abbreviations

ASA, American Society of Anesthesiologists; ASRA, American Society of Regional Anesthesia; BMI, body mass index; CO_2_, carbon dioxide; CSF, cerebrospinal fluid; EtCO_2_, end-tidal carbon dioxide; HR, heart rate; MAP, mean arterial pressure; RASS, Richmond agitation-sedation scale; RR, respiratory rate; SpO_2_, peripheral capillary oxygen saturation; vs, *versus*.

## References

[B1] Ahmer W. Imtiaz S. Alam D. M. Ahmed K. Sajid B. Yousuf J. (2024). Remimazolam *versus* propofol for sedation in gastrointestinal endoscopy and colonoscopy within elderly patients: a meta-analysis of randomized controlled trials. Eur. J. Clin. Pharmacol. 80 (4), 493–503. 10.1007/s00228-024-03624-6 38261005

[B2] Barbosa E. C. Espírito Santo P. A. Baraldo S. Meine G. C. (2024). Remimazolam *versus* propofol for sedation in gastrointestinal endoscopic procedures: a systematic review and meta-analysis. Br. J. Anaesth. 132 (6), 1219–1229. 10.1016/j.bja.2024.02.005 38443286

[B3] Borkett K. M. Riff D. S. Schwartz H. I. Winkle P. J. Pambianco D. J. Lees J. P. (2015). A phase IIa, randomized, double-blind study of remimazolam (CNS 7056) *versus* midazolam for sedation in upper gastrointestinal endoscopy. Anesth. Analgesia 120 (4), 771–780. 10.1213/ANE.0000000000000548 25502841

[B4] Chong S. E. Mohd Nikman A. Saedah A. Wan Mohd Nazaruddin W. H. Kueh Y. C. Lim J. A. (2017). Real-time ultrasound-guided paramedian spinal anaesthesia: evaluation of the efficacy and the success rate of single needle pass. Br. J. Anaesth. 118 (5), 799–801. 10.1093/bja/aex108 28510752

[B5] Elsharkawy H. Maheshwari A. Babazade R. Perlas A. Zaky S. Mounir-Soliman L. (2017). Real-time ultrasound-guided spinal anesthesia in patients with predicted difficult anatomy. Minerva Anestesiol. 83 (5), 465–473. 10.23736/S0375-9393.16.11610-4 28094482

[B6] Forster A. Gardaz J. P. Suter P. M. Gemperle M. (1980). Respiratory depression by midazolam and diazepam. Anesthesiology 53 (6), 494–497. 10.1097/00000542-198012000-00010 7457966

[B7] Grau T. Leipold R. W. Fatehi S. Martin E. Motsch J. (2004). Real-time ultrasonic observation of combined spinal-epidural anaesthesia. Eur. J. Anaesthesiol. 21 (1), 25–31. 10.1017/s026502150400105x 14768920

[B8] Guo J. Qian Y. Zhang X. Han S. Shi Q. Xu J. (2022). Remimazolam tosilate compared with propofol for gastrointestinal endoscopy in elderly patients: a prospective, randomized and controlled study. BMC Anesthesiol. 22 (1), 180. 10.1186/s12871-022-01713-6 35689208 PMC9185932

[B9] Hansen T. G. Engelhardt T. (2025). Remimazolam in children: a comprehensive narrative review. Anesthesiol. Perioper. Sci. 3 (1), 7. 10.1007/s44254-025-00090-w

[B10] Kim S. H. Fechner J. (2022). Remimazolam - current knowledge on a new intravenous benzodiazepine anesthetic agent. Korean J. Anesthesiol. 75 (4), 307–315. 10.4097/kja.22297 35585830 PMC9346281

[B11] Kopp S. L. Vandermeulen E. McBane R. D. Perlas A. Leffert L. Horlocker T. (2025). Regional anesthesia in the patient receiving antithrombotic or thrombolytic therapy: American Society of regional Anesthesia and Pain Medicine evidence-based guidelines (fifth edition). Regional Anesth. Pain Med.–2024-105766. 10.1136/rapm-2024-105766 39880411

[B12] Lee A. Shirley M. (2021). Remimazolam: a review in procedural sedation. Drugs 81 (10), 1193–1201. 10.1007/s40265-021-01544-8 34196946

[B13] Lu K. Wei S. Ling W. Wei Y. Ran X. Huang H. (2022). Remimazolam *versus* propofol for deep sedation/anaesthesia in upper gastrointestinal endoscopy in elderly patients: a multicenter, randomized controlled trial. J. Clin. Pharm. Ther. 47 (12), 2230–2236. 10.1111/jcpt.13797 36334013 PMC10100088

[B14] Morimoto Y. (2022). Efficacy and safety profile of remimazolam for sedation in adults undergoing short surgical procedures. Ther. Clin. Risk Manag. 18, 95–100. 10.2147/TCRM.S304556 35140469 PMC8819169

[B15] Pambianco D. J. Borkett K. M. Riff D. S. Winkle P. J. Schwartz H. I. Melson T. I. (2016). A phase IIb study comparing the safety and efficacy of remimazolam and midazolam in patients undergoing colonoscopy. Gastrointest. Endosc. 83 (5), 984–992. 10.1016/j.gie.2015.08.062 26363333

[B16] Pastis N. J. Yarmus L. B. Schippers F. Ostroff R. Chen A. Akulian J. (2019). Safety and efficacy of remimazolam compared with placebo and midazolam for moderate sedation during bronchoscopy. Chest 155 (1), 137–146. 10.1016/j.chest.2018.09.015 30292760

[B17] Pereira E. M. Moraes V. R. Gaya da Costa M. Nascimento T. S. D. Slawka E. Júnior C. G. (2024). Remimazolam vs. propofol for general anaesthesia in elderly patients: a meta-analysis with trial sequential analysis. Eur. J. Anaesthesiol. 41 (10), 738–748. 10.1097/EJA.0000000000002042 39069837 PMC11377052

[B18] Petersen K. U. Schmalix W. Pesic M. Stohr T. (2024). Carboxylesterase 1-Based drug-drug interaction potential of remimazolam: *in-vitro* studies and literature review. Curr. Drug Metab. 25 (6), 431–445. 10.2174/0113892002308233240801104910 39108117

[B19] Prommer E. (2020). Midazolam: an essential palliative care drug. Palliat. Care Soc. Pract. 14, 2632352419895527. 10.1177/2632352419895527 32215374 PMC7065504

[B20] Provenzano D. A. Hanes M. Hunt C. Benzon H. T. Grider J. S. Cawcutt K. (2025). ASRA pain medicine consensus practice infection control guidelines for regional anesthesia and pain medicine. Regional Anesth. pain Med.–2024-105651. 10.1136/rapm-2024-105651 39837579

[B21] Reves J. G. Fragen R. J. Vinik H. R. Greenblatt D. J. (1985). Midazolam: pharmacology and uses. Anesthesiology 62 (3), 310–324. 10.1097/00000542-198503000-00017 3156545

[B22] Rex D. K. Bhandari R. Desta T. DeMicco M. P. Schaeffer C. Etzkorn K. (2018). A phase III study evaluating the efficacy and safety of remimazolam (CNS 7056) compared with placebo and midazolam in patients undergoing colonoscopy. Gastrointest. Endosc. 88 (3), 427–437.e6. 10.1016/j.gie.2018.04.2351 29723512

[B23] Sessler C. N. Gosnell M. S. Grap M. J. Brophy G. M. O'Neal P. V. Keane K. A. (2002). The Richmond agitation-sedation scale: validity and reliability in adult intensive care unit patients. Am. J. Respir. Crit. Care Med. 166 (10), 1338–1344. 10.1164/rccm.2107138 12421743

[B24] Sneyd J. R. (2023). Remimazolam – current status, opportunities and challenges. Anesthesiol. Perioper. Sci. 1 (3), 25. 10.1007/s44254-023-00021-7

[B25] Vallejo M. C. Kumaraswami S. Zakowski M. I. (2024). American society of anesthesiologists 2023 guidance on neurologic complications of neuraxial analgesia/anesthesia in obstetrics. Anesthesiology 140 (6), 1235–1236. 10.1097/ALN.0000000000004967 38578973

[B26] Zaporowska-Stachowiak I. Szymański K. Oduah M. T. Stachowiak-Szymczak K. Łuczak J. Sopata M. (2019). Midazolam: safety of use in palliative care: a systematic critical review. Biomed. and Pharmacother. = Biomedecine and Pharmacother. 114, 108838. 10.1016/j.biopha.2019.108838 30981104

[B27] Zhou Y. Hu P. Jiang J. (2017). Metabolite characterization of a novel sedative drug, remimazolam in human plasma and urine using ultra high-performance liquid chromatography coupled with synapt high-definition mass spectrometry. J. Pharm. Biomed. Analysis 137, 78–83. 10.1016/j.jpba.2017.01.016 28104560

