# Supplementary material for: Remimazolam used for sedation during spinal anesthesia puncture provides faster sedation effect and increase recovery rate compared to midazolam: a randomized controlled trial
Source: Front Pharmacol. 2025 Oct 20;16:1685312. doi: 10.3389/fphar.2025.1685312 (PMC12580118; doi:10.3389/fphar.2025.1685312)
Supplement: Supplementary file 1 [file Table1.docx]

**Supplementary Table 1: Specific reasons for crowd exclusion.**

| **Exclusion Category** | **Cases (n, %)** |
| --- | --- |
| **Contraindications** | 26 (55.3%) |
| Coagulopathy | 12 (25.5) |
| Severe depression | 3 (6.4%) |
| Obstructive sleep apnea | 10 (21.3) |
| Infection at puncture site | 1 (2.1%) |
| **Declined to participate** | 6 (12.8%) |
| **Investigator decision** | 15 (31.9%) |
| Poor communication ability | 5 (10.6) |
| Participation in another clinical trial | 3 (6.4%) |
| Difficulty in follow-up | 7 (14.9) |
| **Total** | **47 (100.0%)** |
